# Supplementary figures and images for: Structural spine plasticity: Learning and forgetting of odor-specific subnetworks in the olfactory bulb
Source: PLoS Comput Biol. 2022 Oct 24;18(10):e1010338. doi: 10.1371/journal.pcbi.1010338 (PMC9632792; doi:10.1371/journal.pcbi.1010338)

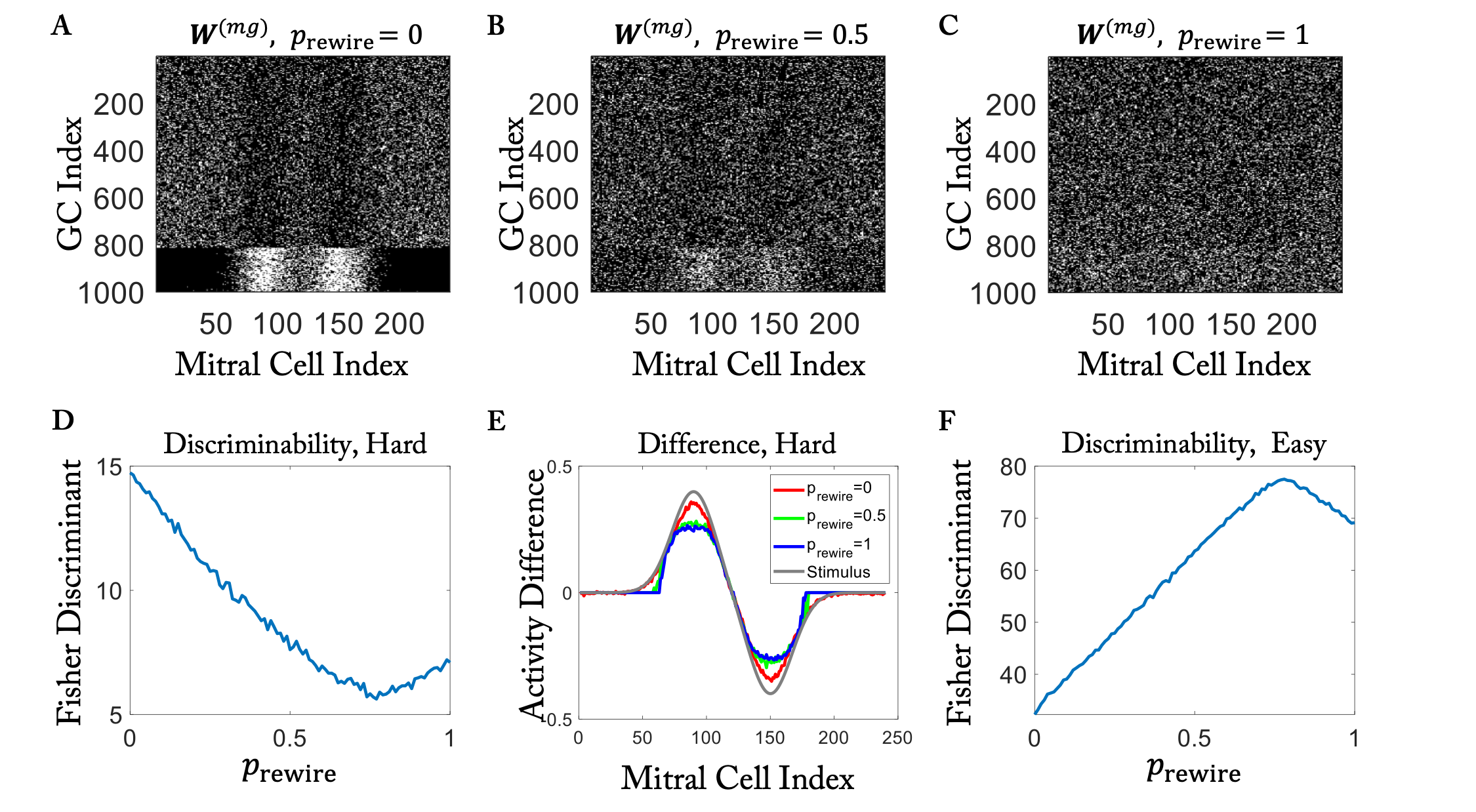

Supplement: S1 Fig — (A to C) Connectivity matrices after rewiring a network that was trained with the hard task (Fig 3C, bottom). Rewiring probability prewire = 0, 0.5, 1, respectively. (D) For the hard task discriminability predominantly decreases with increasing rewiring probability prewire. (E) Activity differences are reduced after rewiring. (F) For the easy task discriminability predominantly increases with increasing prewire (cf. Fig 3C, top). (TIFF) [file pcbi.1010338.s001.tiff]

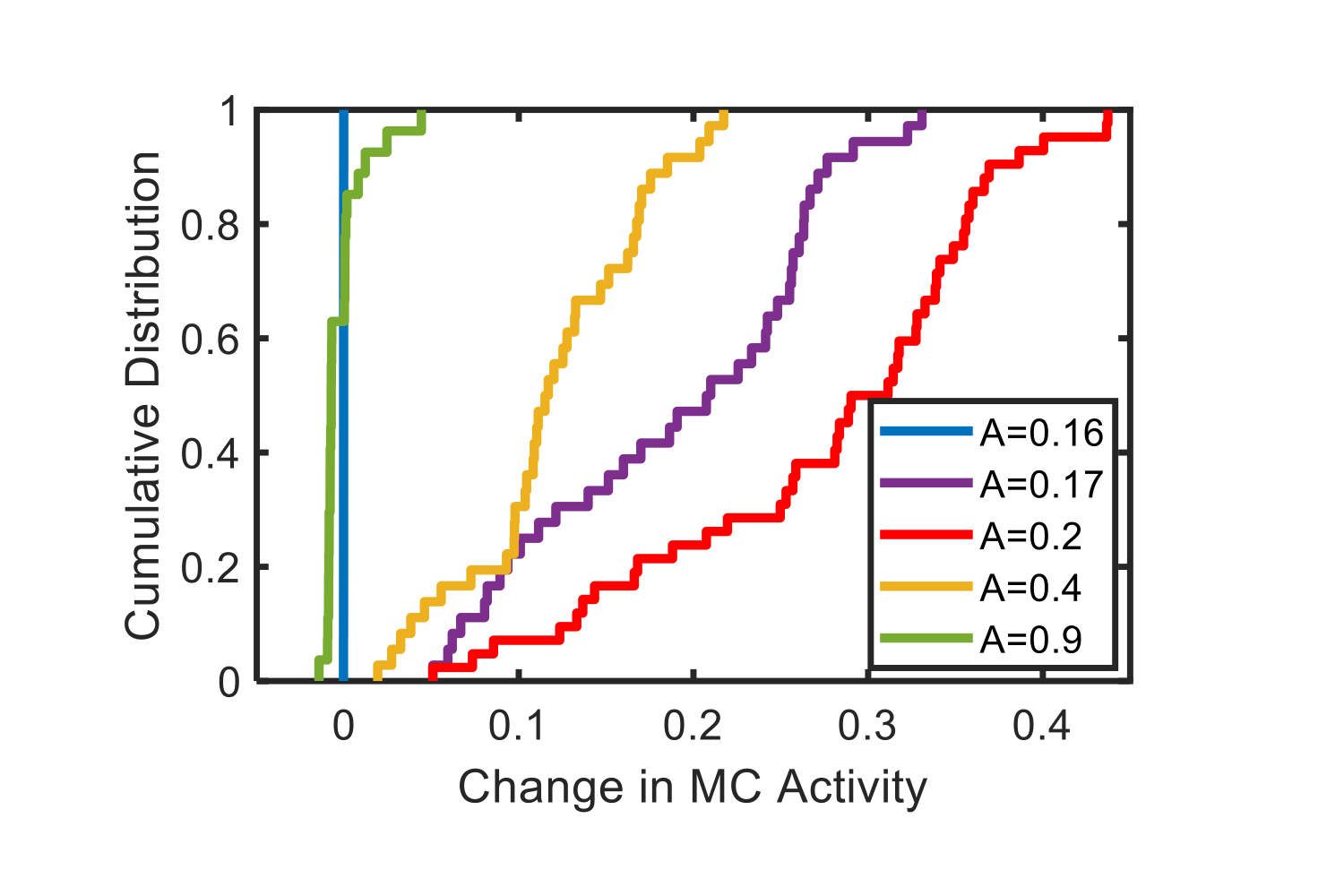

Supplement: S2 Fig — CDF for the increase in the MC response to odor A due to training with that odor at a reduced amplitude 2A after the network has been pre-trained with that odor at amplitude 2. For protocol see Fig 7. (TIFF) [file pcbi.1010338.s002.tiff]

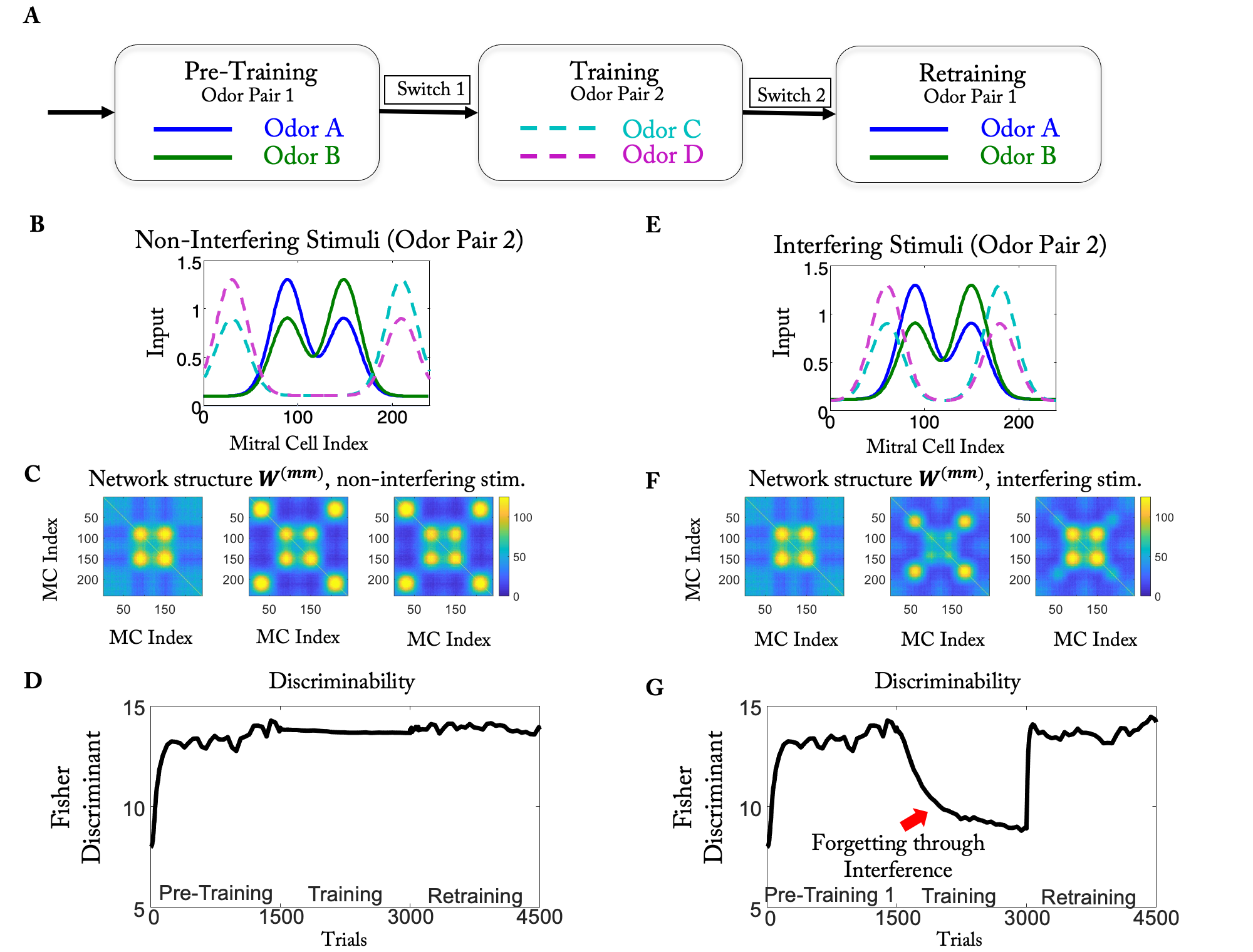

Supplement: S3 Fig — (A) Training protocol. (B) Non-interfering stimuli for odor pair 2. (C) Connectivities after pre-training, training, and retraining, respectively. (D) Discriminability is retained throughout the training. (E to G) as (B to D) but for interfering stimuli. During the training the network forgets most of the previously learned structure. Retraining recovers the previous performance. (TIFF) [file pcbi.1010338.s003.tiff]

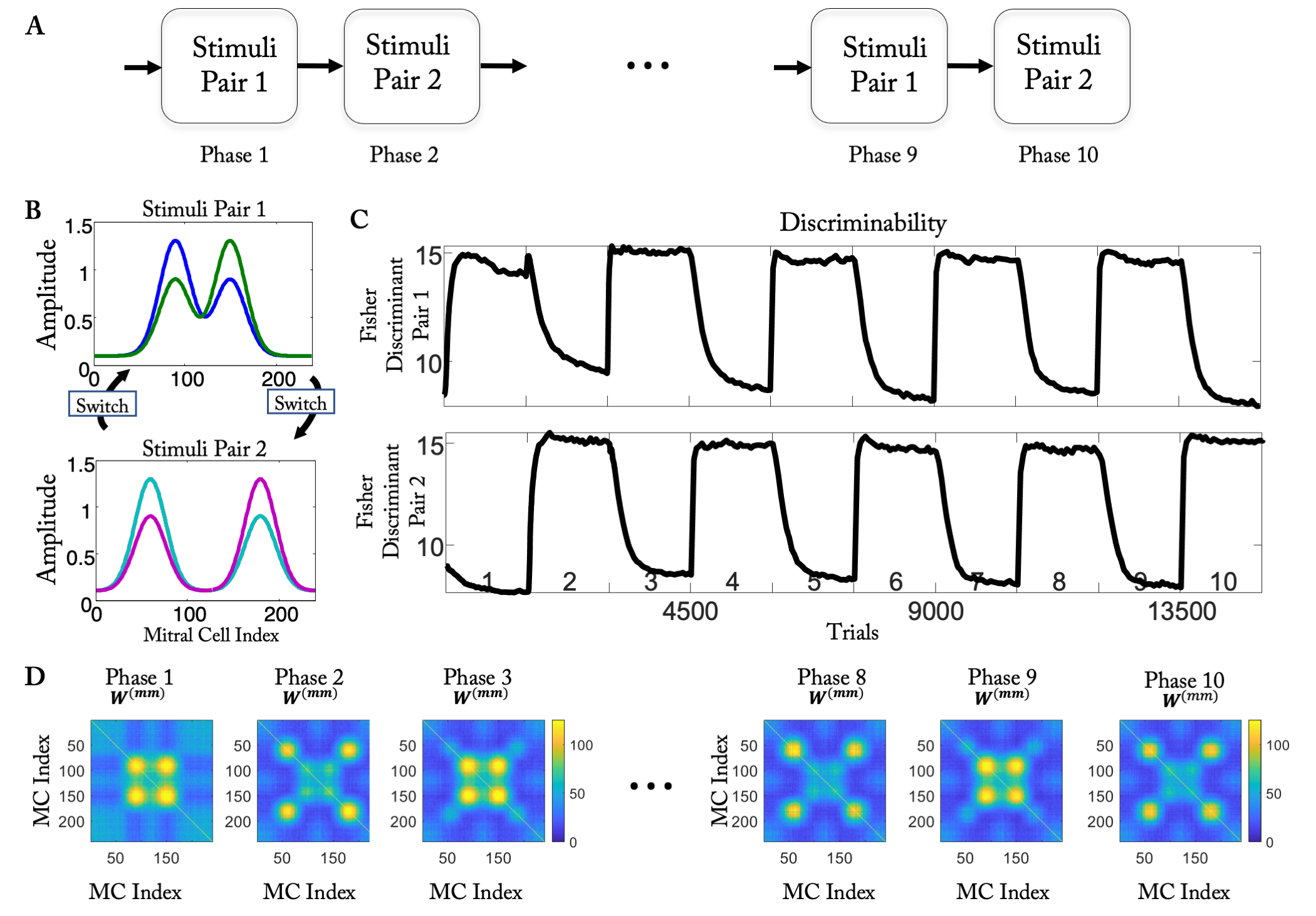

Supplement: S4 Fig — (A) Expanding the training protocol of S3A Fig to 10 phases. (B) (Top) Stimuli of odor pair 1. (Bottom) Interfering stimuli of odor pair 2. (C) The Fisher discriminant of odor pair 1 (top) and pair 2 (bottom). Learning (increasing Fisher discriminant) proceeds faster than forgetting (decreasing Fisher discriminant). The numbers above the x-axis indicate the learning phase. (D) Effective connectivity W(mm) alternates. (TIFF) [file pcbi.1010338.s004.tiff]

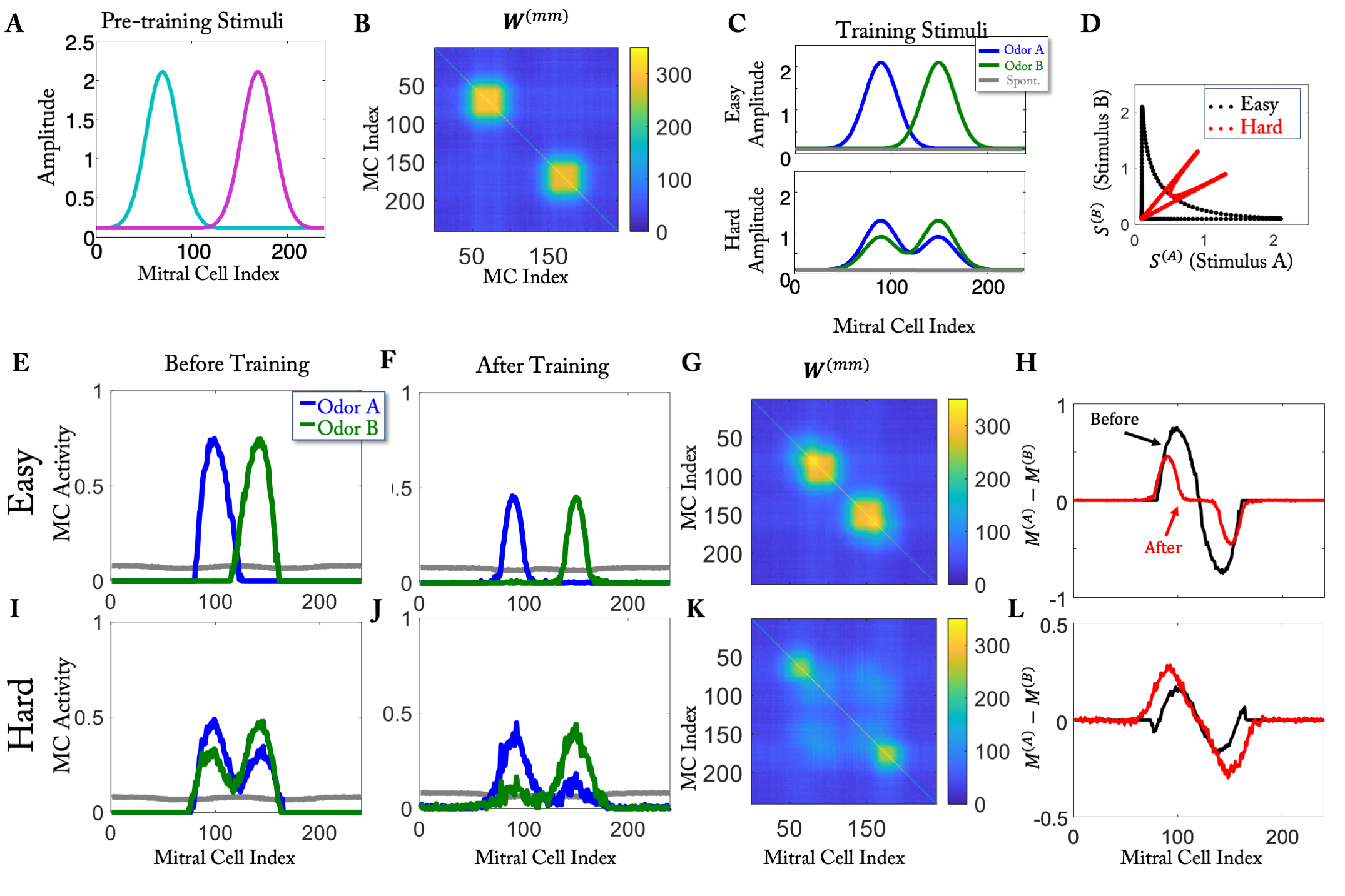

Supplement: S5 Fig — Here γ = 1.7 ⋅ 10−3. Training with dissimilar stimuli reduces their discriminability (E to H), whereas training with very similar enhances their discriminability (I to L) (cf. Fig 3). (TIFF) [file pcbi.1010338.s005.tiff]

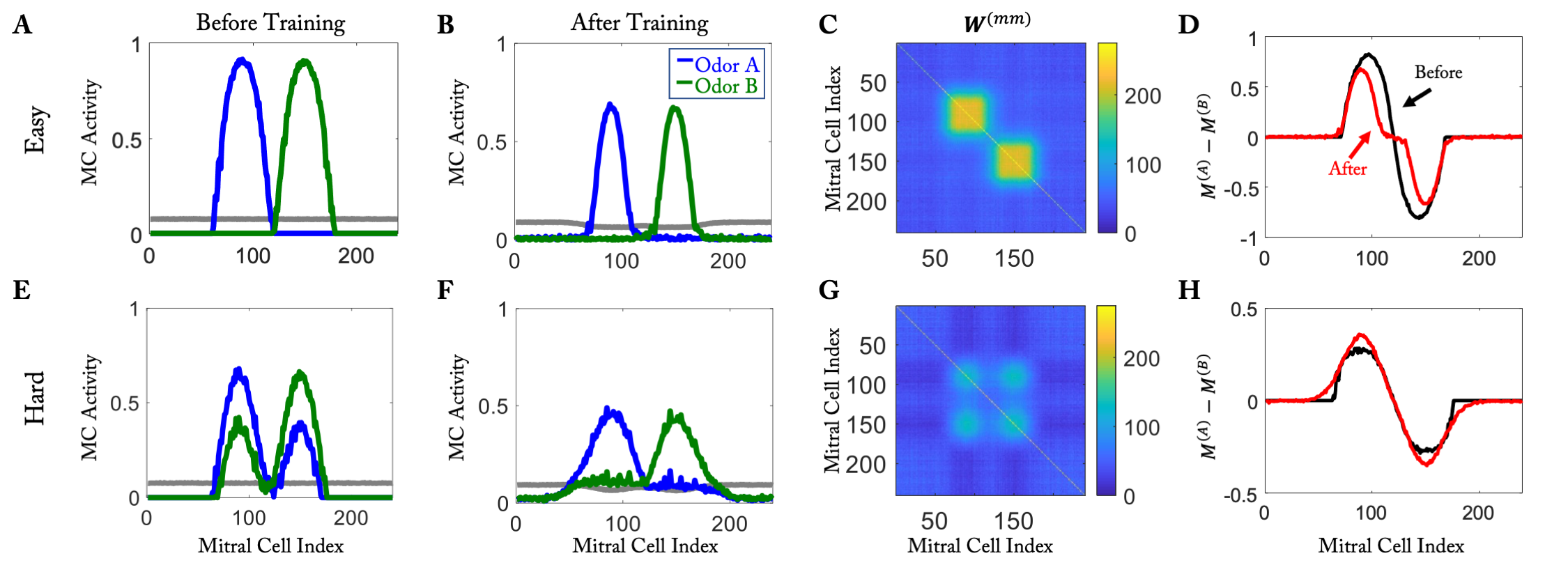

Supplement: S6 Fig — The training starts with a random homogeneous network (cf. Fig 3). (TIFF) [file pcbi.1010338.s006.tiff]

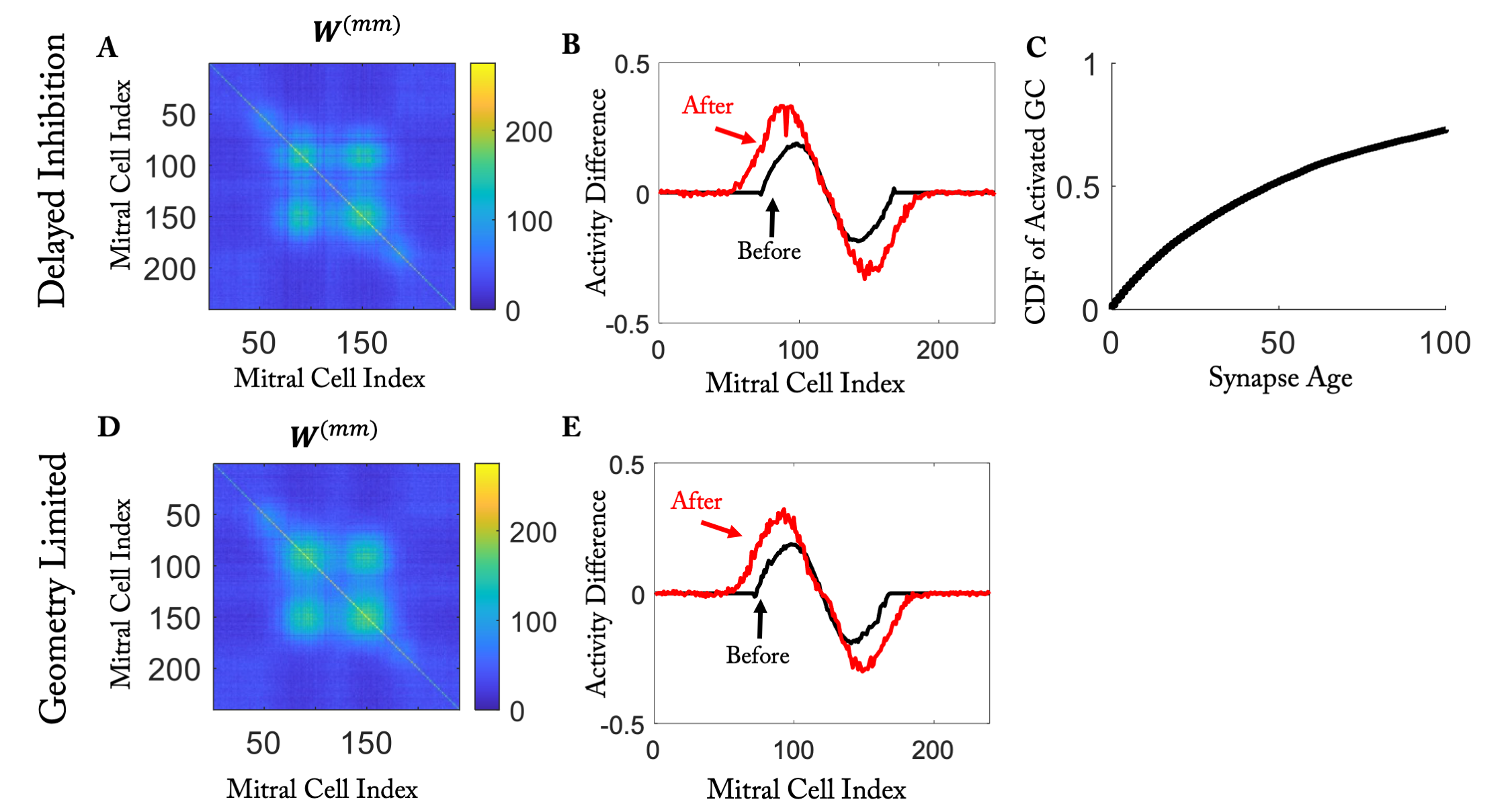

Supplement: S7 Fig — (A–C) The results of setting maturation of the inhibitory synapses lagged 2 timesteps. (A) The effective connectivity after training. (B) Activity difference before and after training. (C) The cumulative distribution function of synapse age. When the lag is 2, only a limited fraction of connections does not inhibit MCs. (D, E) The results when the inhibitory synapses have a geometry limitation. Organized as (A, B). (TIFF) [file pcbi.1010338.s007.tiff]

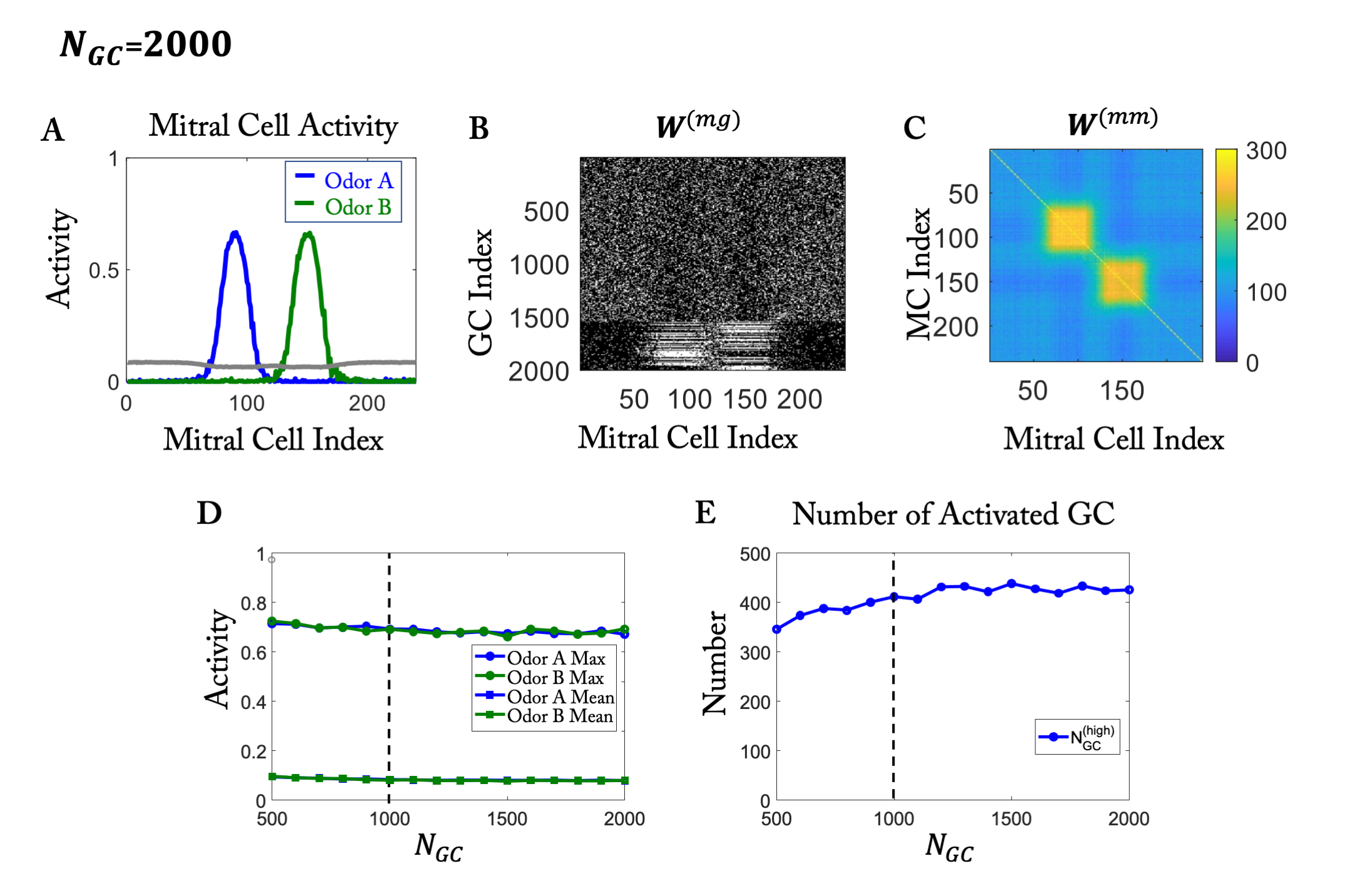

Supplement: S8 Fig — (A-C) Training with simplified easy stimuli as in Fig 1D and random initial network as in Fig 1F, but with twice as many GCs. (A) MC activity after training (cf. Fig 1H). (B) Connectivity W(mg) after training. (C) Effective connectivity W(mm). Compared to Fig 1J the connectivity is not quite as selective. (D) The maximal and mean MC activity after training as a function of the number of GCs. (E) The number of activated GCs (G > G(1)) depends only weakly on the total number of GCs. In (D, E) the vertical dashed line marks the value used in the rest of the paper. (TIFF) [file pcbi.1010338.s008.tiff]

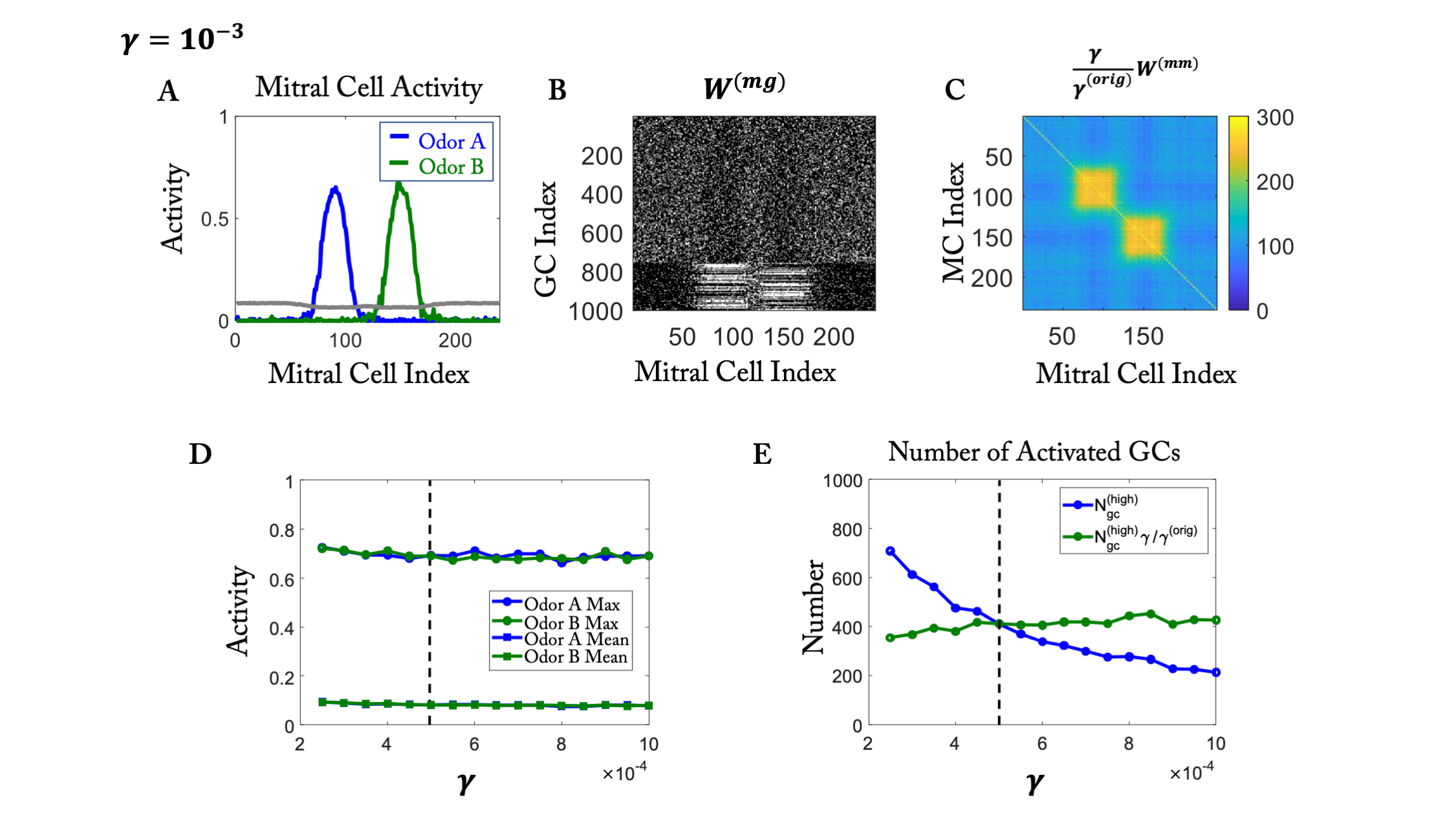

Supplement: S9 Fig — The results are organized as in S8 Fig. (C) The connectivity is slightly less selective than in Fig 1J. (D,E) The vertical dashed line indicates the value used in the rest of the paper. (D, E) To allow a direct comparison, W(mm) and NGC(high) have been re-scaled by γγ(orig) with γ(orig) = 5 × 10^−4^. (TIFF) [file pcbi.1010338.s009.tiff]

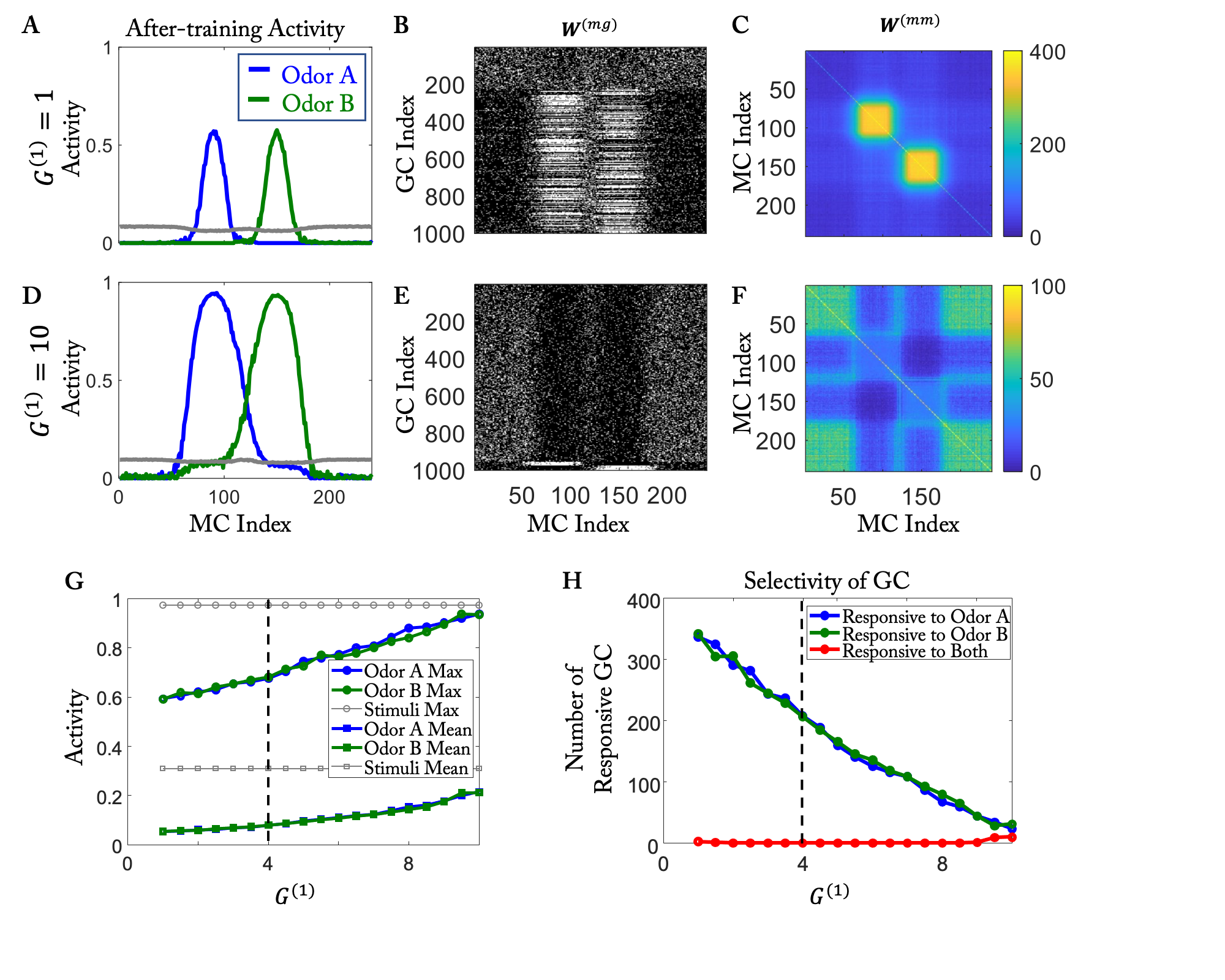

Supplement: S10 Fig — (A,B,C) Results for G(1) = 1. (A) MC activity after training. (B) Connectivity W(mg) between MCs and GCs. (C) Effective connectivity W(mm). (D,E,G) as (A,B,C) except for G(1) = 10. Activated MCs are connected with fewer GCs (compare E with B), resulting in weaker disynaptic inhibition (compare F with C) and higher MC activity (compare D to A). In (F) most of the GCs cannot reach the high threshold. As a result, the synapses that connect to strongly activated MCs are removed faster than those connecting to weakly activated MCs. Thus, the effective connectivity among the activated MCs is lower than the background. (G) Maximal and mean MC activity increase with increasing G(1). The gray lines indicate the corresponding values without inhibition from GCs (by setting γ = 0). (H) The number of responsive GC decreases with increasing G(1). (G,H) The vertical dashed line indicates the value used in the rest of the paper. (TIFF) [file pcbi.1010338.s010.tiff]

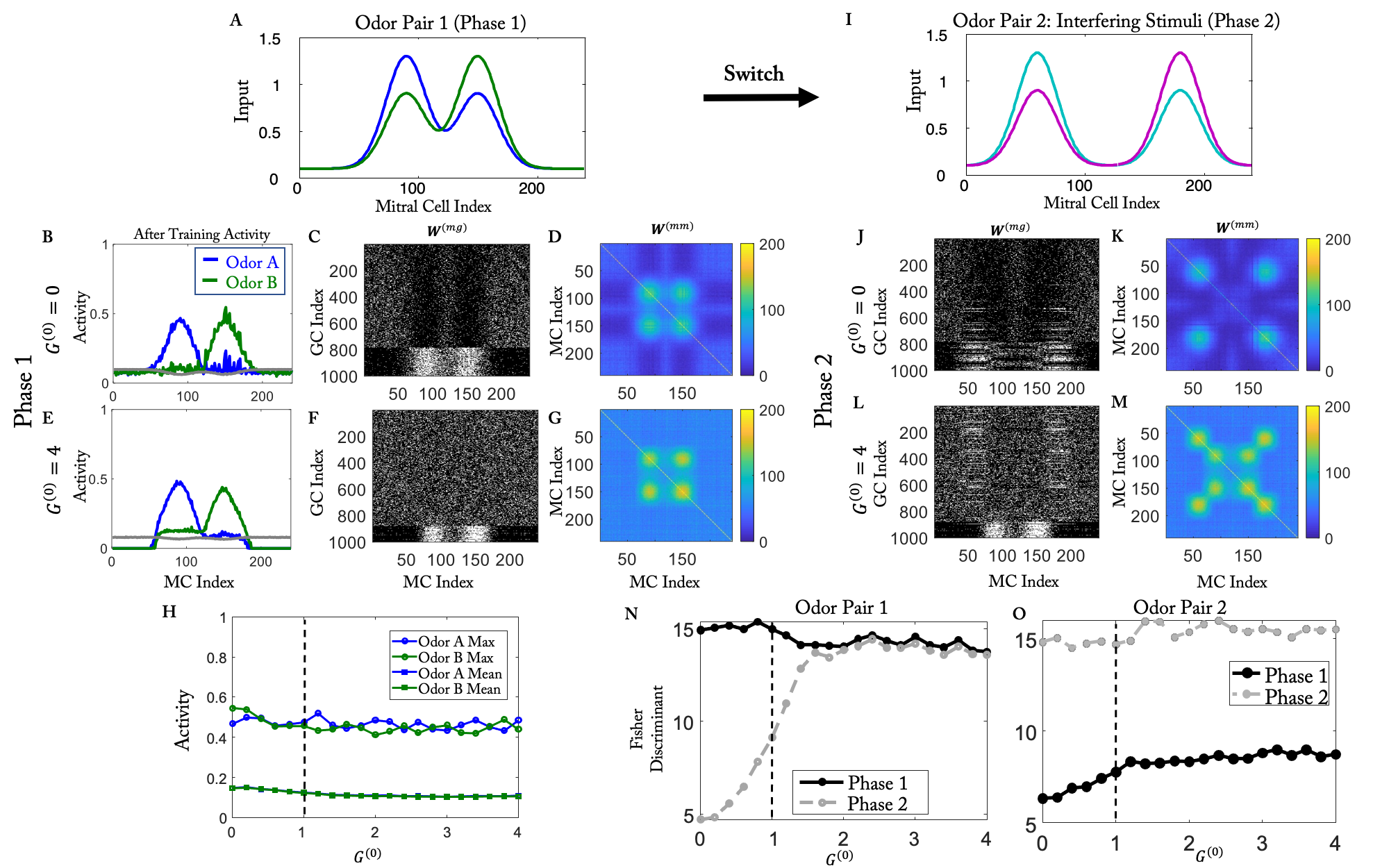

Supplement: S11 Fig — (A-G) Phase 1. (A) Training stimuli in phase 1. (B) MC activity after training. (C) Connectivity W(mg). (D) Effective connectivity W(mm). (E to G) as (B to D) except for G(0) = 4. (H) Maximal and mean MC activity after training in phase 1 as a function of G(1). (I to M) as (A to G) but after Phase 2. (I) Interfering training stimuli. (J-M) For G(0) = 0 the network forgets the previously learned connectivity, but not for G(0) = 4. (N) The Fisher discriminant for odor pair 1 is unaffected by G(0) in phase 1, but substantially reduced in phase 2 for small G(0). (O) For odor pair 2 the Fisher discriminant depends only little on G(0). The vertical dashed lines in (H,N,O) indicate the value used in the rest of the paper. (TIFF) [file pcbi.1010338.s011.tiff]

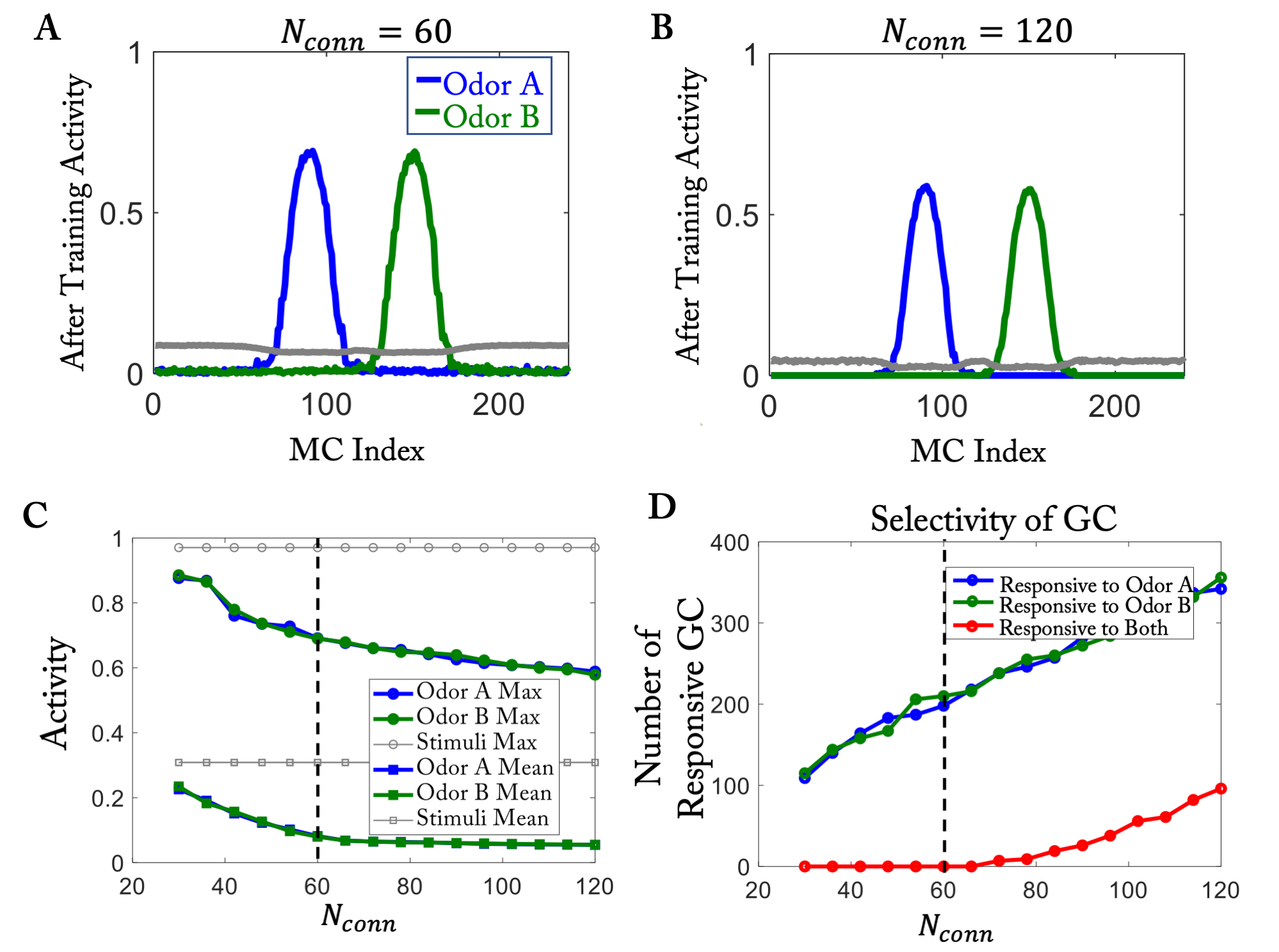

Supplement: S12 Fig — Easy stimuli as in Fig 1D with random initial network as in Fig 1F. The ratio of maximal to initial number of connections is kept fixed at k/Nconn ≡ 1.1. (A, B) The MC activity is very similar for Nconn = 60 and Nconn = 120. (C) Maximal and mean MC activity decrease with increasing Nconn. The gray lines indicate the corresponding values without inhibition from GCs (by setting γ = 0). (D) The selectivity of the GCs is impaired for Nconn > 60: a population of cells emerges that respond to both odors (red line). (TIFF) [file pcbi.1010338.s012.tiff]

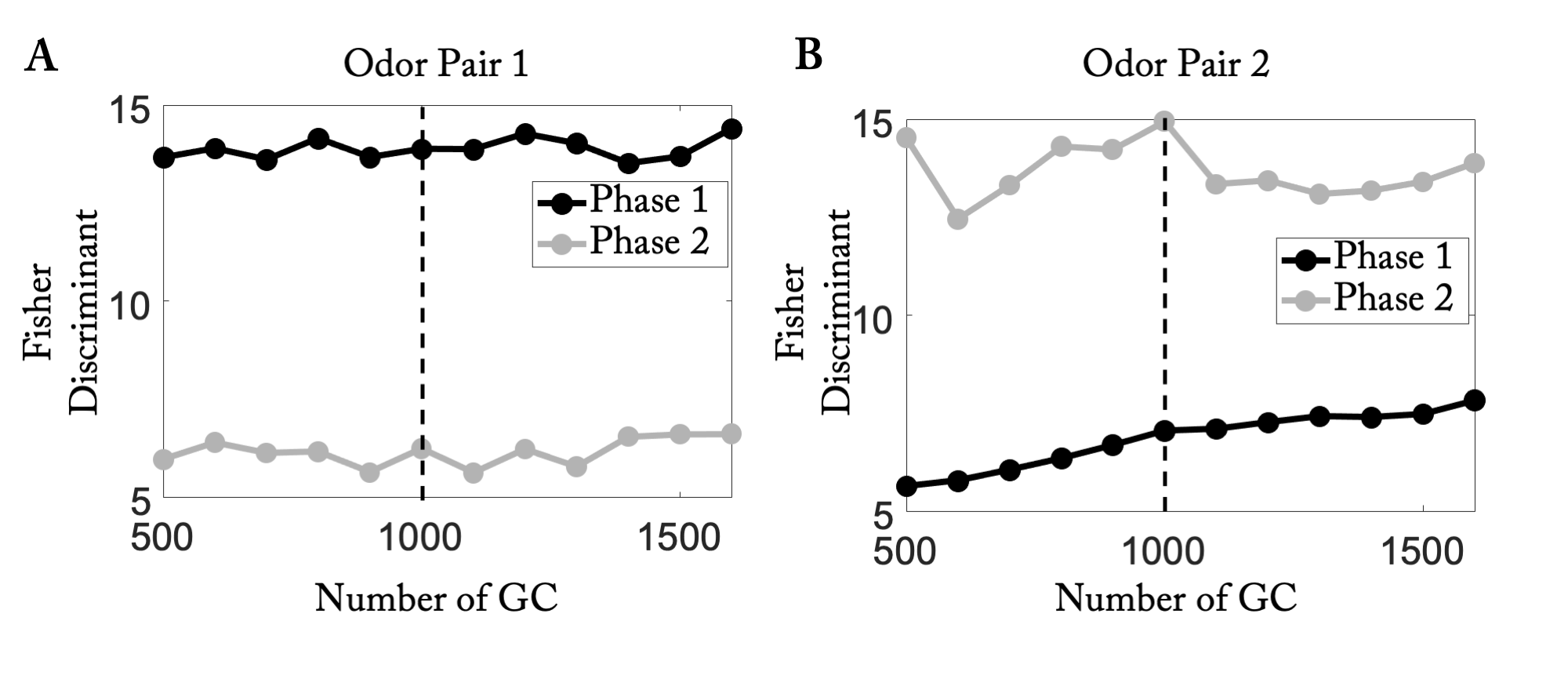

Supplement: S13 Fig — Independent of the number of GCs, the discriminability of odor pair 1, which is learned during phase 1 (black line in panel A), is substantially reduced by the subsequent training during phase 2 (gray line in panel A). The discriminability of odor pair 2 that is reached after training with those odors during phase 2 also does not depend on the number of GCs. Only the learning of odor pair 2 during the training with odor pair 1 is slightly improved in the larger network. (G(0) = 1, cf. S11 Fig. N,O). (TIFF) [file pcbi.1010338.s013.tiff]

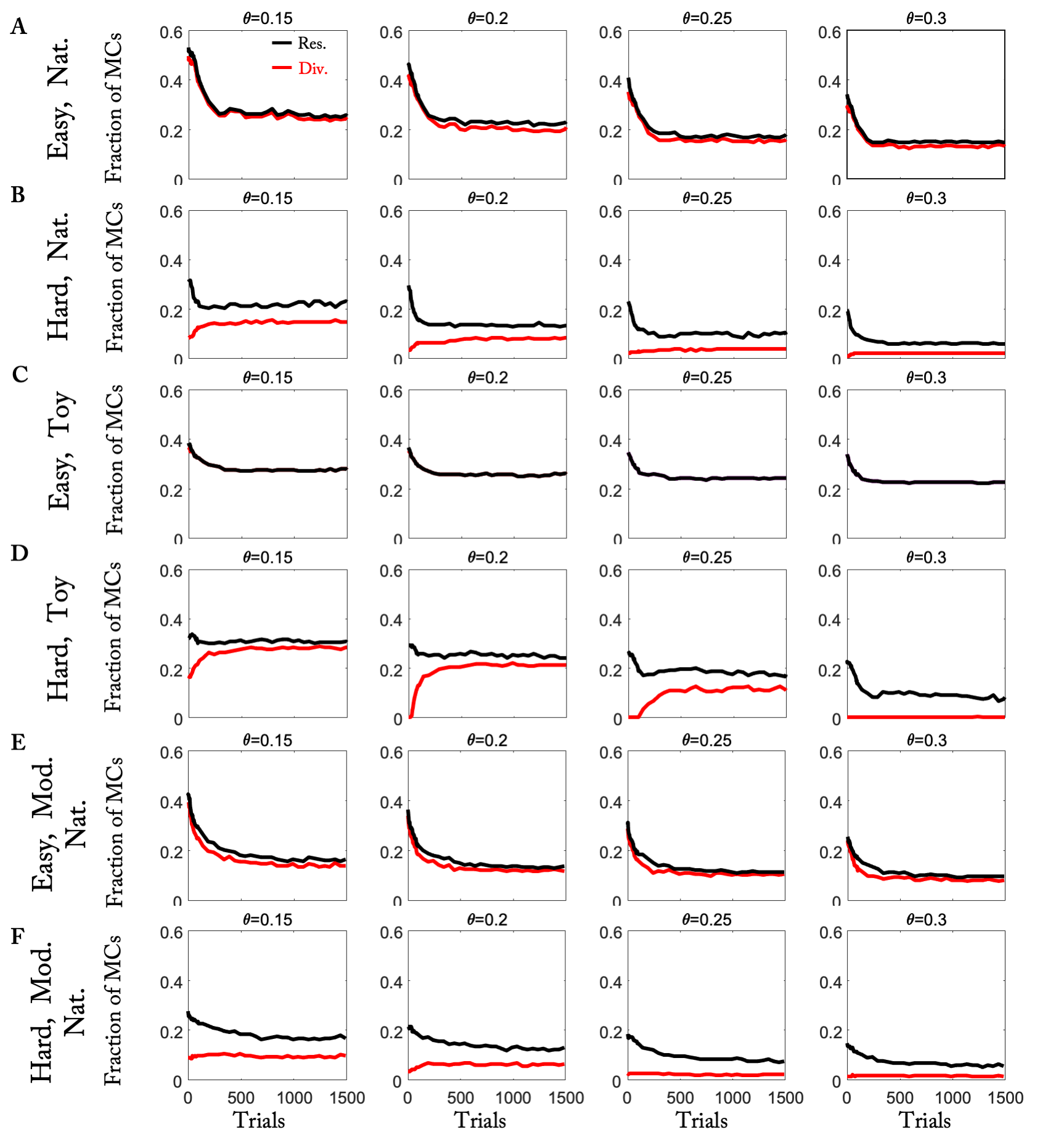

Supplement: S14 Fig — (A, B) Training with naturalistic stimuli for different thresholds θ. The results in Figs 2 and 9 are based on θ = 0.2. (C, D) as (A, B) but for training with simplified stimuli. (E, F) as (A, B) but in the resource-pool model. (TIFF) [file pcbi.1010338.s014.tiff]
