# Supplementary material for: Structural spine plasticity: Learning and forgetting of odor-specific subnetworks in the olfactory bulb
Source: PLoS Comput Biol. 2022 Oct 24;18(10):e1010338. doi: 10.1371/journal.pcbi.1010338 (PMC9632792; doi:10.1371/journal.pcbi.1010338)
Supplement: S1 Text — (PDF) [file pcbi.1010338.s015.pdf]

Randomized Connectivity

To further illustrate that the network learned not by simply changing the number of synapses, but by developing a specific, stimulus-dependent connectivity, we assessed the performance of the network when the connections of each GC were rewired to random MCs with probability p_{rewire} while keeping the number of synapses on each GC the same. For $p_{rewire} = 0$ (S1 Fig A), the connection was the originally learned network; for $p_{rewire} = 1$, the connection for each GC was totally random (S1 Fig C). Indeed, for the highly similar training odors, the discriminability predominantly decreased as the randomness is increased (S1 Fig D). For the network trained on the dissimilar odors (easy task), the discriminability increased, as expected, when the randomness was increased (S1 Fig F). Interestingly, when P_{rewire} was increased beyond ≈ 0.7 these trends reversed (S1 Fig D and F) somewhat.
