# Supplementary material for: Structural spine plasticity: Learning and forgetting of odor-specific subnetworks in the olfactory bulb
Source: PLoS Comput Biol. 2022 Oct 24;18(10):e1010338. doi: 10.1371/journal.pcbi.1010338 (PMC9632792; doi:10.1371/journal.pcbi.1010338)
Supplement: S2 Text — (PDF) [file pcbi.1010338.s016.pdf]

Spines are Removed for Intermediate GC Activation

Reactivating GCs at intermediate levels induces the removal of spines (cf. Fig.7 C and D), while reactivation at small or large amplitudes has only little impact on the spine persistence (Fig.7 B and E). This leads to an increase in the mean activity of the corresponding MCs for intermediate reactivation but not for weak or strong reactivation (Fig.7 F). The large shift in the cumulative distribution function for the change in the MC activity (S2 Fig) that arises only for intermediate reactivation suggests that this should be observable for a large fraction of the MCs.
