# Supplementary material for: Structural spine plasticity: Learning and forgetting of odor-specific subnetworks in the olfactory bulb
Source: PLoS Comput Biol. 2022 Oct 24;18(10):e1010338. doi: 10.1371/journal.pcbi.1010338 (PMC9632792; doi:10.1371/journal.pcbi.1010338)
Supplement: S3 Text — (PDF) [file pcbi.1010338.s017.pdf]

Forgetting due to Interference

The behavior of the model depended strongly on the similarity of the two odor pairs (S3 Fig B and E). When the MCs that were activated during the training did not overlap with those activated in the pre-training, the network preserved the previously learned structure (S3 Fig C). However, if there was significant overlap, the learning of the new stimuli interfered with the previously learned structure and that odor pair was forgotten during the training (S3 Fig F, middle panel). It was re-learned by re-training (S3 Fig F, right panel). The different evolution for non-interfering and for interfering stimuli is reflected in the Fisher discriminant $\mathcal{F}^{(1,2)}$, which is given by the sum over the squares of the d'_i of the individual MCs in response to stimulus pair 1 and 2, respectively (see Methods). The Fisher discriminant $\mathcal{F}^{(1)}$ remained high during the training with odor pair 2 if that pair was non-interfering (S3 Fig D), while it decreased if odor pair 2 was interfering (S3 Fig G). We expect that animals will spontaneously discriminate the odors in a pair only if their bulbar representations are sufficiently different, i.e., if $\mathcal{F}$ is sufficiently large. Thus, the model predicts that at the end of the training phase of a multi-phase perceptual learning task an animal will not spontaneously discriminate any more the odors it learned during pre-training if the training odors interfere with those used in the pre-training. However, for sufficiently different odor pairs, the spontaneous discrimination should remain intact.
