# Supplementary material for: Structural spine plasticity: Learning and forgetting of odor-specific subnetworks in the olfactory bulb
Source: PLoS Comput Biol. 2022 Oct 24;18(10):e1010338. doi: 10.1371/journal.pcbi.1010338 (PMC9632792; doi:10.1371/journal.pcbi.1010338)
Supplement: S4 Text — (PDF) [file pcbi.1010338.s018.pdf]

Rapid Relearning

Repeatedly switching between odor pairs did not impair the learning ability (S4 Fig). In addition, the model predicts that re-learning during re-training is faster than the initial learning during pre-training and forgetting is slower than either of the learning processes.
