# Supplementary material for: Structural spine plasticity: Learning and forgetting of odor-specific subnetworks in the olfactory bulb
Source: PLoS Comput Biol. 2022 Oct 24;18(10):e1010338. doi: 10.1371/journal.pcbi.1010338 (PMC9632792; doi:10.1371/journal.pcbi.1010338)
Supplement: S5 Text — (PDF) [file pcbi.1010338.s019.pdf]

Resource Pool and Simplified Stimuli

The model with resource pool (S5 Fig) exhibited qualitatively the same behavior as the top-k model for the easy and the hard task using simplified stimuli (cf. Fig.3).
