# Supplementary material for: Structural spine plasticity: Learning and forgetting of odor-specific subnetworks in the olfactory bulb
Source: PLoS Comput Biol. 2022 Oct 24;18(10):e1010338. doi: 10.1371/journal.pcbi.1010338 (PMC9632792; doi:10.1371/journal.pcbi.1010338)
Supplement: S6 Text — (PDF) [file pcbi.1010338.s020.pdf]

On the Impact of Pre-Training

In Fig.3 the network was pre-trained on a set of stimuli that partially overlapped with the stimuli used in the training itself. This pre-training is not essential for the different outcome of the discriminability of the easy and the hard stimuli. Even without pre-training the easy stimuli became less discriminable after training, while the hard stimuli became more discriminable (S6 Fig).
