# Supplementary material for: Structural spine plasticity: Learning and forgetting of odor-specific subnetworks in the olfactory bulb
Source: PLoS Comput Biol. 2022 Oct 24;18(10):e1010338. doi: 10.1371/journal.pcbi.1010338 (PMC9632792; doi:10.1371/journal.pcbi.1010338)
Supplement: S7 Text — (PDF) [file pcbi.1010338.s021.pdf]

Robustness of the Model I

To demonstrate the robustness of the model, we first assessed the impact of some modifications of the model.

For the formation of the reciprocal synapses little is known about the timescales of the maturation of the excitatory synapses relative to that of the inhibitory synapses. In the main part of the paper we assumed that inhibition becomes functional at the same as the excitation. Here, we tested if the model behaves differently if inhibitory synapses mature later than the excitatory synapses. If the inhibitory synapses become only functional 2 computational time steps after the excitatory synapses, the model performs similarly (S7 Fig A-C). Since 100 timesteps in our model correspond very roughly to about 1 day training in the experiments, a lag of 2 timesteps would correspond to a lag on the order of 30 minutes. However, the performance deteriorates if the lag is increased and the model fails for lags of 10 or larger. A detailed analysis of this break-down is beyond the scope of this study.

While action potentials can propagate along the secondary dendrites of MCs and can drive excitatory synapses on spines even far from the soma, the GC-driven inhibition originating from those spines is likely to affect the MC somata only within a quite limited spatial range. To assess the impact of this spatial limitation, we blocked the inhibition in half of the MC-GC pairs: while in all spines the MC$\rightarrow$GC connections excited the GCs, only half of the MC$\leftarrow$GC connections in those spines were effective in inhibiting the MCs. To make the total amount of inhibition comparable, we doubled the number of granule cells. With this modification, the results are similar to the original model (S7 Fig D, E).
