# Supplementary material for: Structural spine plasticity: Learning and forgetting of odor-specific subnetworks in the olfactory bulb
Source: PLoS Comput Biol. 2022 Oct 24;18(10):e1010338. doi: 10.1371/journal.pcbi.1010338 (PMC9632792; doi:10.1371/journal.pcbi.1010338)
Supplement: S8 Text — (PDF) [file pcbi.1010338.s022.pdf]

Robustness of the Model II

Further, we tested the influence of various parameters on the model performance. Changing the total number N_{GC} of GCs affected the performance very little, since the number of GCs that were strongly activated and connected to specific MCs did not change significantly, which influenced the effective connectivity $W^{(mm)}$ only mildly (S8 Fig).
