# Supplementary material for: Structural spine plasticity: Learning and forgetting of odor-specific subnetworks in the olfactory bulb
Source: PLoS Comput Biol. 2022 Oct 24;18(10):e1010338. doi: 10.1371/journal.pcbi.1010338 (PMC9632792; doi:10.1371/journal.pcbi.1010338)
Supplement: S9 Text — (PDF) [file pcbi.1010338.s023.pdf]

Robustness of the Model III

Analogously, changes in the inhibitory strength γ were largely compensated by a change in the number of activated GCs (S9 Fig). This feedback stabilizing the overall inhibition arises from the reciprocal character of the MC-GC connections: new connections increase the inhibition of the MCs, which decreases the excitation of the GC and in turn inhibits the formation of new connections.
