# Supplementary material for: Structural spine plasticity: Learning and forgetting of odor-specific subnetworks in the olfactory bulb
Source: PLoS Comput Biol. 2022 Oct 24;18(10):e1010338. doi: 10.1371/journal.pcbi.1010338 (PMC9632792; doi:10.1371/journal.pcbi.1010338)
Supplement: S10 Text — (PDF) [file pcbi.1010338.s024.pdf]

The Connectivity Depends on the Threshold $G^{(1)}$

What does control the overall inhibition, is the threshold $G^{(1)}$ in the activation function $\phi(G)$ (S10 Fig). Only GCs with activity larger than $G^{(1)}$ form more synapses and generate stronger selective inhibition. Thus, for larger $G^{(1)}$ fewer GCs had sufficiently large activity to sustain their synapses, which reduced the overall inhibition and increased the MC activity level. The change in overall inhibition could be associated with a change in the selectivity. To quantify this, we called a GC responsive to odor A (B), if after training its activity surpassed $G^{(1)}$ for odor A (B). Despite the increase in the inhibition, the number of GCs that responded to both odors remained low when decreasing $G^{(1)}$ (S10 Fig H, red line), indicating that the selectivity depended very little on $G^{(1)}$.
