# Supplementary material for: Structural spine plasticity: Learning and forgetting of odor-specific subnetworks in the olfactory bulb
Source: PLoS Comput Biol. 2022 Oct 24;18(10):e1010338. doi: 10.1371/journal.pcbi.1010338 (PMC9632792; doi:10.1371/journal.pcbi.1010338)
Supplement: S11 Text — (PDF) [file pcbi.1010338.s025.pdf]

The Memory Depends on the Threshold $G^{(0)}$

The other threshold in the activation function, $G^{(0)}$, affects the removal but not the formation of synapses. For low values of $G^{(0)}$ weakly activated GCs removed many of their synapses, particularly those connecting them to active MCs (S11 Fig C, D). This lead to higher selectivity of the connectivity and with it to a weaker inhibition of the spontaneously active MCs that were not odor-driven (S11 Fig B, E). It had, however, little impact on the maximal MC amplitudes (S11 FigH). The removal of synapses played a larger role in retaining and forgetting connections. To assess its impact, we trained the model in a second phase with a new pair of stimuli that overlapped with the stimuli learned in phase 1 (S11 Fig I). For $G^{(0)} = 0$ any weak activity $(0 < G < G^{(1)})$ triggered the removal of spines and lead in phase 2 to an almost complete forgetting of the connectivity learned in phase 1 (S11 Fig D, K) and with it to poor discrimination of the previously learned odors (S11 Fig N). However, for large $G^{(0)}$ the previously learned connectivity was maintained (S11 Fig G, M) and with it the discriminability of the previously learned stimuli. The discrimination of the newly learned stimulus pair 2 was, however, not significantly affected by $G^{(0)}$ (S11 Fig O).
