# Supplementary material for: Structural spine plasticity: Learning and forgetting of odor-specific subnetworks in the olfactory bulb
Source: PLoS Comput Biol. 2022 Oct 24;18(10):e1010338. doi: 10.1371/journal.pcbi.1010338 (PMC9632792; doi:10.1371/journal.pcbi.1010338)
Supplement: S12 Text — (PDF) [file pcbi.1010338.s026.pdf]

The Selectivity Depends on the Maximal Number of Connections

The maximal number of connections k that each GC can make had a significant impact on MC amplitudes and on the selectivity of the connections. With increasing k, each GC integrated input from more MCs, and more easily surpassed the threshold $G^{(1)}$. Each of these more activated GCs inhibited a larger number of MCs, increasing the overall inhibition (S12 Fig C). When the maximal number of connections was larger than the number of MCs activated by one of the odors, not all connections of a GC could be made to MCs that were activated by odor A, say. The remaining connections could then be made to MCs corresponding to odor B without destabilizing the other synapses. As a result, GCs that responded to both odors emerged (S12 Fig D).
